# Graphene-Based Cathode Materials for Lithium-Ion Capacitors: A Review

**DOI:** 10.3390/nano11102771

**Published:** 2021-10-19

**Authors:** Dong Sui, Meijia Chang, Zexin Peng, Changle Li, Xiaotong He, Yanliang Yang, Yong Liu, Yanhong Lu

**Affiliations:** 1 Key Laboratory of Function-Oriented Porous Materials, College of Chemistry and Chemical Engineering, Luoyang Normal University, Luoyang 471934, China; pengzexinly@126.com (Z.P.); lichangle77@163.com (C.L.); hexiaotong0928@126.com (X.H.); yangyli@mail.ustc.edu.cn (Y.Y.); 2 School of Environmental Engineering and Chemistry, Luoyang Institute of Science and Technology, Luoyang 471023, China; 3 Collaborative Innovation Center of Nonferrous Metals of Henan Province, Henan Key Laboratory of Non-Ferrous Materials Science & Processing Technology, School of Materials Science and Engineering, Henan University of Science and Technology, Luoyang 471023, China; liuyong209@haust.edu.cn; 4 School of Chemistry & Material Science, Langfang Normal University, Langfang 065000, China

**Keywords:** lithium-ion capacitors, graphene, graphene-based nanomaterials, capacitor-type electrodes, cathode materials

## Abstract

Lithium-ion capacitors (LICs) are attracting increasing attention because of their potential to bridge the electrochemical performance gap between batteries and supercapacitors. However, the commercial application of current LICs is still impeded by their inferior energy density, which is mainly due to the low capacity of the cathode. Therefore, tremendous efforts have been made in developing novel cathode materials with high capacity and excellent rate capability. Graphene-based nanomaterials have been recognized as one of the most promising cathodes for LICs due to their unique properties, and exciting progress has been achieved. Herein, in this review, the recent advances of graphene-based cathode materials for LICs are systematically summarized. Especially, the synthesis method, structure characterization and electrochemical performance of various graphene-based cathodes are comprehensively discussed and compared. Furthermore, their merits and limitations are also emphasized. Finally, a summary and outlook are presented to highlight some challenges of graphene-based cathode materials in the future applications of LICs.

## 1. Introduction

With the increasing global energy consumption, the growing concerns of the depletion of fossil fuels, the corresponding climate change and environmental pollution have arisen [1,2,3]. Thus, clean and renewable energy sources, including solar, wind and tide powers, have become the priority and the most promising choice [4,5,6]. However, the highly effective utilization of all these energy sources is seriously hindered by their intermittent production and uneven geographical distribution [7]. High-performance energy storage materials and devices with characteristics of high energy and power densities, low cost and long-term stability have become a worldwide research hot topic for both academia and industry [8,9,10,11,12]. In addition, the rapid development of consumer electronics and electric vehicles also calls for high-performance energy storage devices [13,14,15]. Consequently, various electrochemical energy storage systems, including the conventional lead-acid battery (LABs) and Ni-metal hydride batteries (NiMHBs), lithium-ion batteries (LIBs), electric double-layer capacitors (EDLCs) and the hybrid device, have been investigated and applied in these booming fields, and their electrochemical performances are summarized in Table 1.

**Figure 1:**
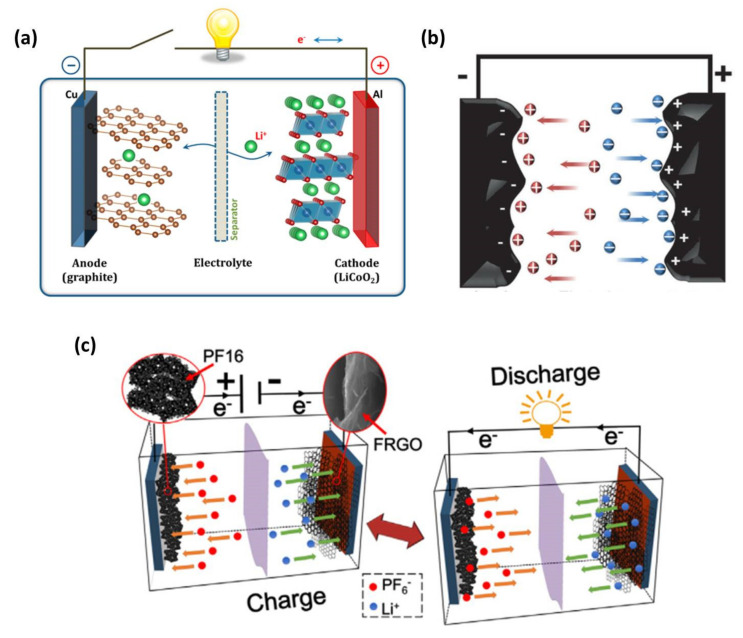
The schematic of the charge/discharge mechanisms of typical (**a**) LIBs, (**b**) EDLCs and (**c**) LICs. ((**a**) Reprinted with permission from Ref. [19]. Copyright 2013 American Chemical Society. (**b**) Reprinted with permission from Ref. [22]. Copyright 2014 Wiley-VCH. (**c**) Reprinted with permission from Ref. [41]. Copyright 2015 Elsevier.).

Obviously, conventional LABs and NiMHBs suffer from relatively low energy density, which is mainly ascribed to the low capacity of the electrode materials and small working voltage limited by the decomposition of aqueous electrolyte. Moreover, the poor power output ability and short cycling life further restrain their applications. Compared with traditional energy storage devices, there is no doubt that LIBs and EDLCs are the two most important systems nowadays and have more competitive advantages [11,16,17,18]. Owing to their different energy storage mechanisms, LIBs and EDLCs demonstrate sharply different electrochemical properties. As demonstrated in Figure 1a, LIBs store or release energy via lithium ions’ intercalation into/de-intercalation from the bulk electrode materials with the removal and addition of electrons through the external circuit [19]. This is the reason why LIBs are also called “rocking chair batteries”. Hence, LIBs usually deliver much higher energy density than other systems because of the bulk Faradaic reaction [20,21]. On the other hand, EDLCs, which employ two identical porous carbon-based electrodes as the anode and cathode (Figure 1b), have the features of excellent power density and long cycle life [22]. The outstanding performances are due to the electrostatic, non-Faradaic and physical double-layer charge-discharge mechanism, which stores/releases energy through extremely rapid adsorption/desorption of electrolyte ions at the electrode/electrolyte interface [23,24]. However, both LIBs and EDLCs still face insurmountable challenges, inhibiting their wide applications in fields where high energy and power as well as excellent cycling life are all required [25,26,27,28,29]. For example, the high energy barrier of redox reaction, sluggish Li^+^ diffusion rate in the bulk electrode and low electrical conductivity of the cathode lead to inferior power density, while the continuous and usually uncontrollable decomposition of electrolytes and the serious deterioration of material structure result in a poor cycle life for LIBs [19,30,31,32]. As for EDLCs, their low working voltage (typically lower than 3.0 V) and limited capacity due to the physically electrostatic adsorption/desorption of electrolyte ions on the surface of electrode materials bring about unsatisfactory energy density [33,34]. Therefore, it is highly urgent to develop advanced energy storage devices that could combine the advantages of LIBs and EDLCs to fulfill the ever-increasing requirements.

Energy density and power density are two key parameters for advanced energy storage systems. Generally, the energy density (E) and average power density (P_a_) of a device could be calculated based on Equations (1) and (2), respectively [19,35,36]:

E = *∫V*d*q* (1)

P_a_ = E/*t* (2)

where *V* is the maximum working voltage, *q* is the specific capacity and *t* is the discharge time. Clearly, the energy and power densities are largely determined by the capacity and the operation voltage of the device. For the case of EDLCs and hybrid devices, in particular, the energy density and power density could also be expressed by Equations (3) and (4) [36,37]:

E = 1/2 *CV*^2^ (3)

P_max_ = *V*^2^/(4*mR*) (4)

where P_max_, *C*, *m* and *R* are the maximum power density, gravimetric specific capacitance (mainly based on the mass of the anode and cathode), mass of the electrode materials and equivalent series resistance, respectively. As a result, developing high-capacity electrode materials, expanding the operation voltage and reducing the overall impedance are typically applied to obtain high-energy and high-power energy storage systems.

Based on the above discussion, current commercial energy storage systems have their individual drawbacks, and it is still a great challenge to develop a device that could combine the advantages of batteries and EDLCs. Fortunately, lithium-ion capacitors (LICs, also known as lithium-ion hybrid supercapacitors) are emerging as one of the most promising candidates to bridge the performance gap between LIBs and EDLCs by integrating high energy density, high power output, large working voltage and long cycling life into one device [12,38]. Typically, LICs are composed of one battery-type electrode as the anode to provide high energy, and one capacitor-type electrode as the cathode to ensure high power and cycling stability [39,40]. Much different from LIBs and EDLCs, LICs show a hybrid energy storage mechanism. As displayed in Figure 1c, during the charge process, anions (i.e., PF_6_^−^) are adsorbed onto the surface of the capacitor-type electrode with the increase in the cathode potential while the lithium ions from the electrolyte simultaneously intercalate into the battery-type electrode with the decrease in the anode potential [41]. For the discharge process, the anions and the Li^+^ ions leave the cathode and anode, respectively, and return to the electrolyte to release the stored energy. Furthermore, as the cathode and anode work in different potential regions, a much higher working voltage can be obtained for LICs than EDLCs, and thus the energy density can be improved [42]. Overall, LICs have a hybrid charge storage mechanism based on the novel device configuration. The high energy and power densities and long cycling life contributed by the combined merits of the battery-type anode from LIBs and capacitor-type cathode from EDLCs make them an attractive alternative for next-generation energy storage devices.

Since the first protype was proposed by Amatucci et al. using activated carbon (AC) as a cathode and Li_4_Ti_5_O_12_ (LTO) as a battery-type anode [43], the studies of electrolytes, electrode materials and pre-lithiation technology for LICs have obtained great progress, especially for the anode and cathode materials’ design [44,45,46,47]. Considering that the energy and power densities are proportional to the square of the operational voltage, lithium salt-containing organic electrolytes are commonly applied to provide a wide working voltage window [45]. On the other hand, there are also works that adopt aqueous solution as an electrolyte considering the advantages of low cost, environmental friendliness and ease of handling [48,49]. However, they suffer from inferior energy density due to the low working voltage. As for the cathode materials, similar to the requirements of electrodes for EDLCs, the ideal cathode materials should have characteristics of a large specific surface area (SSA), high electrical conductivity and satisfied chemical and electrochemical stability. These materials are mainly porous carbons, including AC, carbon nanotubes (CNTs), biomass-derived carbons and graphene-based materials, etc. [35,50,51]. In general, commercial AC and biomass-derived porous carbon suffer from low SSA and low conductivity, while CNTs tend to form agglomeration because of their high aspect ratio and strong π–π interaction. By contrast, the high SSA, excellent conductivity, adjustable porosity and rich surface chemistry of graphene and graphene-based composites grant them high capacity and outstanding power output, making them the most competitive capacitor-type electrodes for LICs. Unlike the cathodes limited to porous carbonaceous materials, a variety of materials are available to be chosen for the anodes [46,52,53]. In general, anode materials can be divided into three categories based on the different lithium storage mechanisms: intercalation-type materials (e.g., carbonaceous materials, Ti-/Nb-based materials) [22,54,55,56,57,58,59], conversion-type materials (e.g., metal oxide, phosphide or sulfide) [60,61,62,63,64,65,66] and alloying-type materials (e.g., Si-/Sn-based materials) [67,68,69,70]. Nevertheless, anode materials face the challenges of sluggish redox reaction (due to their poor conductivity and low lithium ion diffusion rate) and structural instability (due to the volume change during the charge/discharge process). Consequently, nanostructure engineering and coating with highly conductive carbon materials are typically applied to overcome these drawbacks [71,72]. Moreover, the imbalances in specific capacity, kinetics and structural stability between the anode and cathode result in unsatisfactory energy and power densities and poor long-term stability, seriously limiting their applications [32,73]. Generally, the energy density of the reported LICs falls in the range of 100–200 Wh kg^−1^ (based on the mass of active materials), corresponding to 20–100 Wh kg^−1^ based on the mass of the full device [74]. Obviously, this value is still far less than that of LIBs. It should be pointed out that the limited capacity of the cathode is the primary factor for the unsatisfied overall energy density of LICs. Moreover, well-matched specific capacity between the cathode and anode could also largely improve the overall energy density of LICs. Consequently, it is very imperative to explore high-capacity cathode materials.

**Figure 2:**
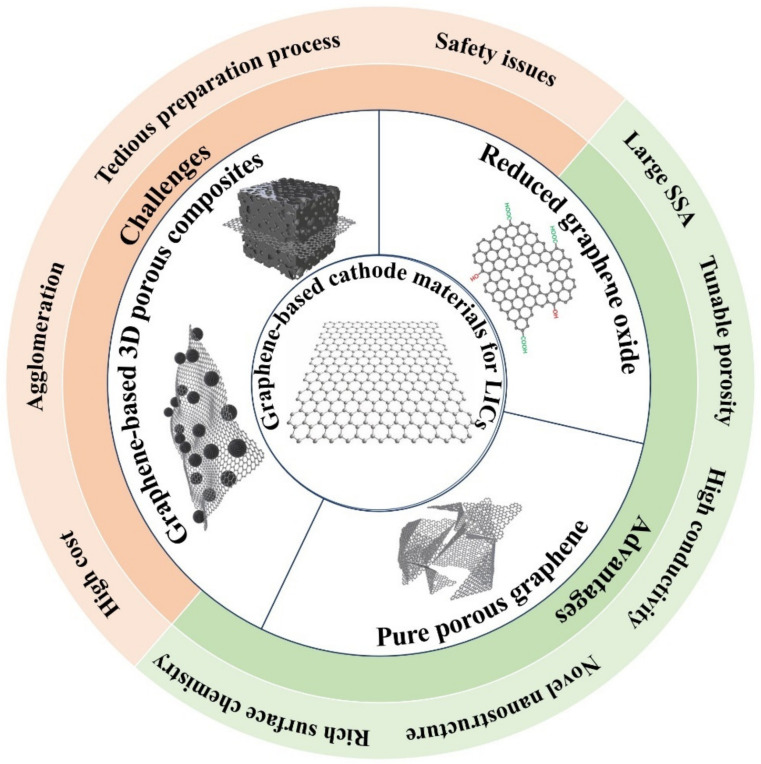
Typical graphene-based cathode materials for LICs and their advantages and the remaining challenges.

Graphene, as a novel two-dimensional (2D) nanocarbon material, has many outstanding characteristics such as high theoretical SSA, astonishing electrical conductivity, tunable porosity and rich surface chemistry [36]. It should be noted that graphene has comparable or even superior properties to other nanocarbon-based materials, making it an excellent candidate either as a high-performance active material or as an attractive flexible support to load other materials for applications in LIBs, SCs and hybrid devices [75,76,77,78]. In particular, graphene-based nanomaterials have been verified as desired capacitor-type cathodes for LICs [37,79]. According to the theoretical calculation, the specific capacitance of graphene can reach as high as 550 F g^−1^ based on the fully used SSA, significantly higher than that of commercial AC and other porous carbon materials [80]. Moreover, the abundant edges, in-plane defects and large number of exposed surface atoms endow graphene with more electrochemical active sites for ion sorption/desorption [81]. Thus, graphene and its composites demonstrate great appeal as capacitor-type electrodes with high capacity in LICs. As demonstrated in Figure 2, graphene or reduced graphene oxide can be directly used as active materials by rationally regulating the structure and surface chemistry. Simultaneously, they can also serve as excellent substrates or building blocks to form 3D porous composites, leading to improved electrical conductivity and/or SSA of the obtained composites. Exciting achievements of graphene-based cathodes in LICs have been made, strongly demonstrating their potential in enhancing the performance of capacitor-type cathodes [36,37]. Accordingly, some reviews about graphene-based anode materials for LICs have been reported [42,79,81,82]. However, reviews that comprehensively cover the development of graphene-based capacitor-type electrode materials are still absent. In this review, recent progresses of graphene-based cathode materials for LICs are systematically summarized, and the roles of graphene are specially emphasized, i.e., high-capacity active materials, conductive supports and building blocks. Furthermore, a summary and outlook are tentatively presented and the remaining challenges in LICs are also discussed.

## 2. Reduced Graphene Oxide as a Cathode Material

### 2.1. Reduced Graphene Oxide

**Figure 3:**
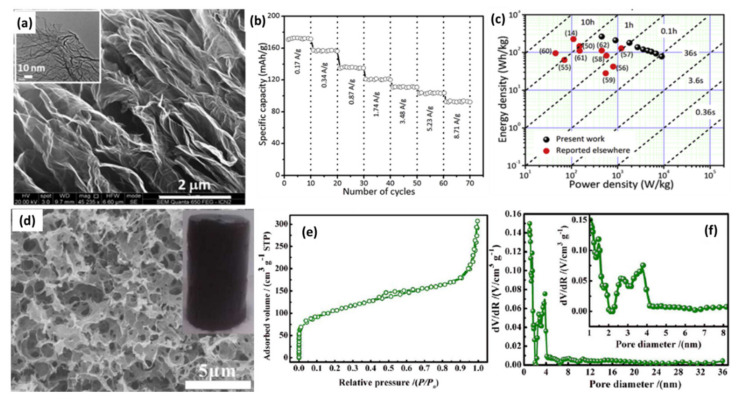
(**a**) FESEM of PRGO (inset shows TEM image); (**b**) Rate capability of PRGO; (**c**) Ragone plot for N-CNPipes//PRGO LIC and other devices. (Reprinted with permission from Ref. [89]. Copyright 2018 Elsevier.) (**d**) SEM image (The inset is the corresponding picture of the PGM), (**e**) N_2_ sorption isotherm, and (**f**) the pore size distribution curves of PGM. (Reprinted with permission from Ref. [91]. Copyright 2015 Elsevier.).

In the early stage, researchers usually applied reduced graphene oxide (rGO) as the capacitor-type active material for LICs because of its feasible fabrication procedure, controllable size and thickness and ease of handling [83]. In addition, the tunable surface functional groups provide extra capacitance via fast redox reaction [84]. For instance, Lee et al. reported an urea-reduced GO (URGO) cathode for LICs by treating GO with urea [85]. URGO shows a specific capacity of 35 mAh g^−1^, which is about a 35% increase compared with conventional AC. Based on the mechanism analysis, the carbonyl groups in the amide groups are responsible for the enhanced capacity through binding with Li^+^ based on the enolization process (N-C=O + Li^+^ + e^−^ ⟷ N-C-O-Li), which has also been observed in Li-organic batteries [86,87]. The as-fabricated LICs paired with the URGO cathode with a pre-lithiation graphite anode show an energy density of approximately 106 Wh kg^−1^ and power density of 4200 W kg^−1^ based on the mass of active materials, which is not only much higher than that of supercapacitors, but also superior to hydrazine-reduced GO and AC-based devices. Another group assembled LICs with trigol-reduced GO (TRGO) as the cathode and LTO as the anode, and the LTO//TRGO LICs delivered a maximum energy density of 45 Wh kg^−1^ with a stable cycling performance of 5000 times [88]. The improved energy density can be ascribed to the high reversible capacity (58 mAh g^−1^) of the as-synthesized TRGO, which is almost twice that of AC. Dubal et al. reported all nanocarbon LICs using partially reduced graphene oxide (PRGO) as the cathode and N-doped carbon nanopipes (N-CNPipes) as the anode [89]. The as-prepared PRGO shows a highly porous structure with abundant wrinkles on the surface (Figure 3a). It should be noted that PRGO can be easily obtained in large scale just by annealing GO powder at 120 ℃. Furthermore, it demonstrates high specific capacity (171 mAh g^−1^ at 0.17 A g^−1^) and excellent rate capacity (92.3 mAh g^−1^ at 8.71 A g^−1^), making it a superior cathode for LICs (Figure 3b). The super performance of PRGO was attributed to the highly porous, interconnected networks and the partial reduction of graphene oxide. These novel structures contribute to the high electrical conductivity while maintaining a substantial amount of C=O redox groups. The as-fabricated all carbon-based LICs exhibit long and stable cycling, high energy density of 262 Wh kg^−1^ at 450 W kg^−1^ and high power density of 9000 W kg^−1^ at 78 Wh kg^−1^ (Figure 3c). Nevertheless, the above-reported LICs directly adopted chemically reduced GO as cathodes usually suffer from limited specific capacity because of their low SSA, which results from severe agglomeration/restacking of rGO nanosheets [80]. Hence, effective strategies are needed to improve the performance of rGO-based capacitor-type electrodes.

### 2.2. Three-Dimensional Reduced Graphene Oxide

The agglomeration of rGO can be largely suppressed by forming three-dimensional (3D) porous hydrogels or foams through self-assembly technology. Monolithic rGO hydrogel [90], porous graphene macroform (PGM) [91] and graphene grass [92] have been reported via a hydrothermal reduction combined with the freeze-drying process. The as-prepared samples have a 3D porous network structure, which can provide multidimensional transport pathways for electrons and electrolytes and minimize the transport distances between bulk electrodes and electrolytes [90,93]. This enables them to have high capacity and excellent power output, making them highly attractive cathode materials for LICs. Taking the formation of PGM for example, GO was partially reduced during hydrothermal treatment and self-assembled into graphene hydrogel through random cross-linking between flexible graphene sheets [91]. After removing the solvent by the freeze-drying process, the as-prepared PGM demonstrates a cylindrical morphology without obvious shrinkage (inset of Figure 3d). As displayed in Figure 3d, PGM has a continuous 3D porous framework with abundant micropores, mesopores and macropores, which could be verified from the N_2_ adsorption isotherm and pore size distribution (PSD) curves (Figure 3e,f). By paring with a LTO/C anode, the PGM-based LICs show an acceptable energy density of 72 Wh kg^−1^, which still keeps 40 Wh kg^−1^ at a power density of 8.3 kW kg^−1^.

In general, reduced GO has been adopted as a capacitor-type electrode for LICs in early studies due to its facile preparation. For easy comparison, the properties of reduced graphene oxide cathodes and the LICs based on them are demonstrated in Table 2. Overall, rGO-based cathode materials usually display relatively low specific capacity due to the low SSA and thus unsatisfactory energy density. Although rGO-based 3D porous cathode materials deliver improved capacity compared with the pristine rGO because of their highly porous structure, the energy density of the fabricated LICs is still far from the commercial application. Furthermore, the insufficient reduction of rGO results in low electrical conductivity and inferior power density. These drawbacks may be overcome by developing pure graphene-based cathodes with well-defined porous structure, which are also considered to be promising alternatives to reduced GO due to their inherently high conductivity and large SSA. In addition, introducing highly conductive fillers (i.e., CNTs) to obtain 3D porous composites is also considered to be a promising alternative to reduced GO.

## 3. Pure Porous Graphene as a Cathode Material

As descried above, reduced GO-based cathodes often deliver relatively low capacity and poor rate performance, which can be mainly ascribed to the inferior electrical conductivity and low SSA. To overcome these disadvantages, one of the effective strategies is to prepare pure graphene-based porous materials, which can keep the inherently excellent properties of large SSA and high conductivity.

### 3.1. Porous Graphene Prepared with Template

**Figure 4:**
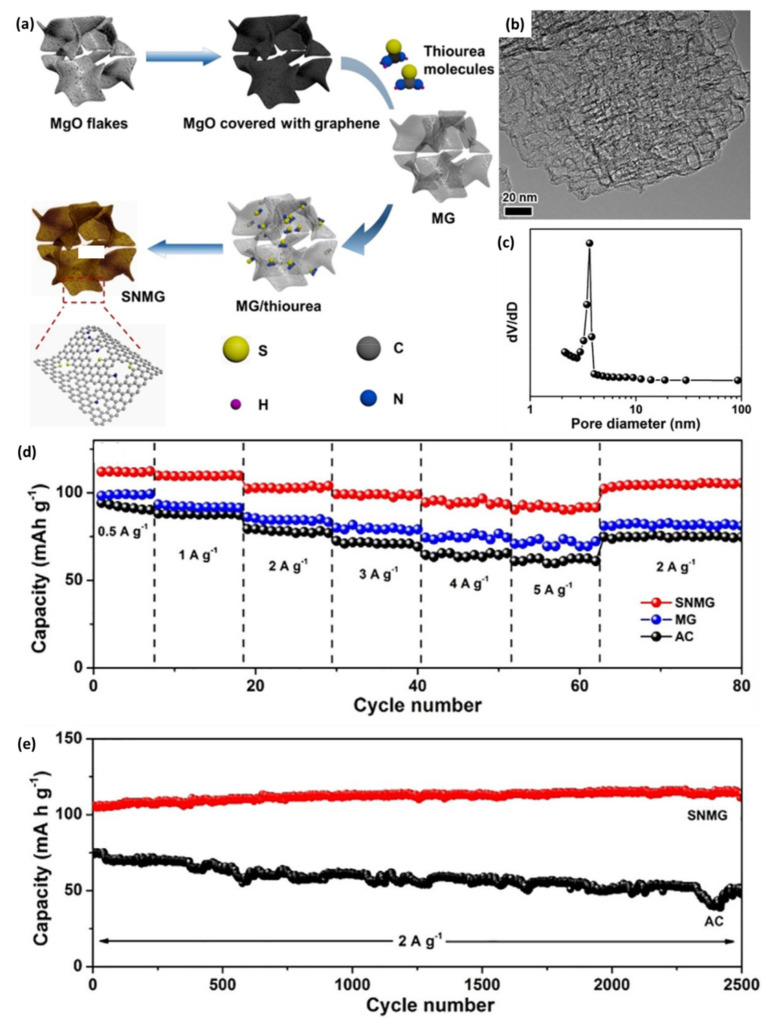
(**a**) Schematic of the preparation process of SNMG; (**b**) TEM image and (**c**) PSD of SNMG; (**d**) Rate capability of SNMG, MG and AC; (**e**) Cycling stability of SNMG and AC. (Reprinted with permission from Ref. [96]. Copyright 2018 Wiley-VCH.).

Typically, the methodology combining chemical vapor deposition (CVD) and hard template is applied to prepare porous graphene materials with high conductivity and large SSA [94,95]. For example, Ma et al. designed S,N-codoped mesoporous graphene (SNMG) based on the CVD method using heavy MgO flakes as a solid template [96]. As displayed in Figure 4a, graphene firstly grew on the surface and pores of MgO thin lamellas by CH_4_ catalytic deposition in a fluidized-bed reactor, and then the templates were removed by acid washing to obtain few-layered mesoporous graphene (MG), followed by annealing with thiourea to form the final products. The as-prepared SNMG exhibits a highly porous structure with pore size in the range of 2–10 nm, which is clearly demonstrated in the TEM image (Figure 4b) and PSD (Figure 4c). The interconnected and controllable mesopores combined with sulfur and nitrogen co-doping endow the SNMG cathode with outstanding rate capability. A reversible capacity of 112 mAh g^−1^ can be achieved at a current density of 0.5 A g^−1^ and still retain 92 mAh g^−1^ at 5 A g^−1^, which is much better than that of MG and AC (Figure 4d). In addition, SNMG shows excellent cycling stability with no obvious capacity decay after 2500 cycles (Figure 4e). As expected, the as-fabricated LICs exhibit exciting electrochemical performance with maximum energy and power densities of 86.2 Wh kg^−1^ and 7443 W kg^−1^ when paired with an LTO anode. Later, the same group also constructed micron-sized porous graphene belts (PGBs) using MgO belts as templates by the CVD approach [97]. Thanks to the unique one-dimensional belt-like architecture, the restacking or aggregation of PGBs is effectively impeded. In addition, the high ratio of length to diameter, high conductivity, large SSA and good structural stability contribute to the superior capacitive behavior of the as-obtained PGBs. Porous graphene prepared by CVD usually has a high-quality structure and relatively less defects compared with GO-derived materials. Therefore, the obtained cathode materials deliver excellent rate performance and superior cycling stability.

### 3.2. Porous Graphene Prepared by Chemical Activation

Chemical activation is an effective and feasible strategy to produce porous carbon materials with high SSA and large pore volume, which is commonly used in the preparation of commercial AC [98]. The activation process with KOH as an activation agent is proposed to follow the below reactions [99]:

6KOH + C → 2K + 3H_2_ + 2K_2_CO_3_ (5)

K_2_CO_3_ → K_2_O + CO_2_ (6)

K_2_CO_3_ + 2C → 2K + 3CO (7)

K_2_O + C → 2K + CO (8)

CO_2_ + C → 2CO (9)

**Figure 5:**
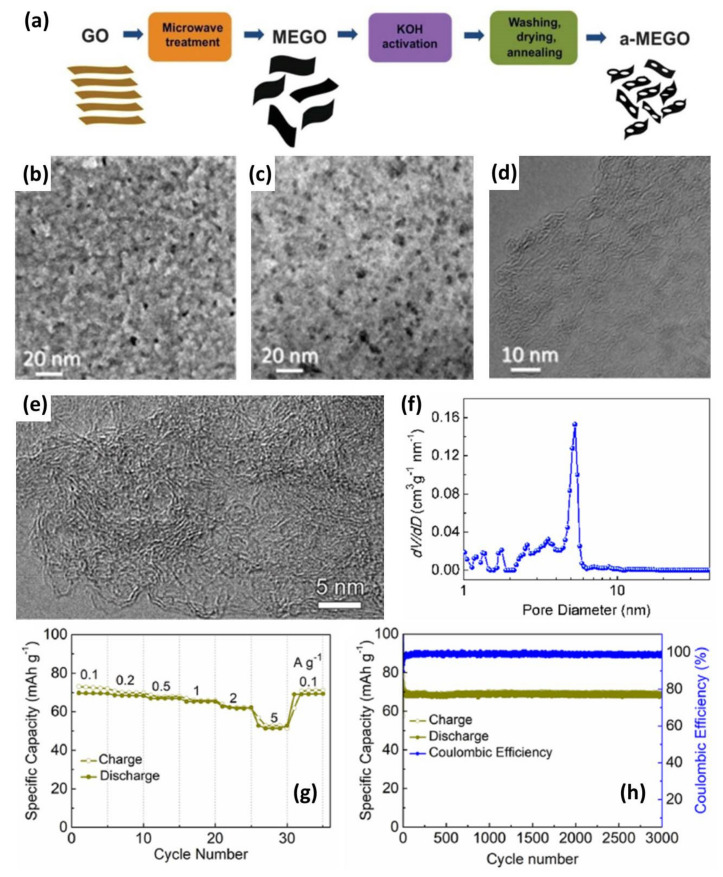
(**a**) Schematic of preparation of a-MEGO. (**b**) High-resolution SEM, (**c**) annular dark field STEM and (**d**) high-resolution TEM images of a-MEGO. (Reprinted with permission from Ref. [100]. Copyright 2011 American Association for the Advancement of Science.) (**e**) High-resolution TEM images and (**f**) pore size distribution curve of PG; (**g**) Rate capability and (**h**) cycling stability of PG. (Reprinted with permission from Ref. [103]. Copyright 2020 Elsevier.).

The activation procedure starts from the reaction of KOH with carbon and then the decomposition of K_2_CO_3_ and/or reactions of K_2_CO_3_/K_2_O/CO_2_ with carbon, creating a porous carbon material. Ruoff et al. reported “activated microwave expanded graphite oxide” (a-MEGO) by irradiating GO in a microwave oven in combination with chemical activation (Figure 5a) [100]. As exhibited in Figure 5b–d, a-MEGO possesses a dense pore structure with a continuous 3D network of highly curved and predominantly atom-thick walls. The extraordinarily high SSA (3100 m^2^ g^−1^) coupled with a continuous 3D network of extremely small size pores (ranging from <1 nm to 10 nm) endowed a-MEGO with a high specific capacity (125 mAh g^−1^) [101]. Specifically, the graphite//a-MEGO LIC yielded an energy density of 147.8 and 53.2 Wh kg^−1^ at an operating potential of 4 V based on the active materials and the packaged full cell, respectively. Inspired by the pioneering work of Ruoff’s group, pure graphene-based porous materials with high SSA and large pore volume were prepared by the chemical activation of reduced GO foam or hydrogel and used as capacitor-type electrodes for LICs [102,103]. For example, porous graphene (PG) reported by Yang et al. showed a highly crumpled and porous structure after hydrothermal reaction and KOH treatment (Figure 5e) [103]. Specifically, PG has a surface area of 2103 m^2^ g^−1^ and pore volume of 1.8 cm^3^ g^−1^ with PSD centered at about 5 nm (Figure 5f). Consequently, the interpenetrated pores and excellent conductivity endowed PG with outstanding electrochemical performance, such as excellent rate capability and good long-term stability (Figure 5g–h).

### 3.3. Porous Graphene Prepared by Other Methods

**Figure 6:**
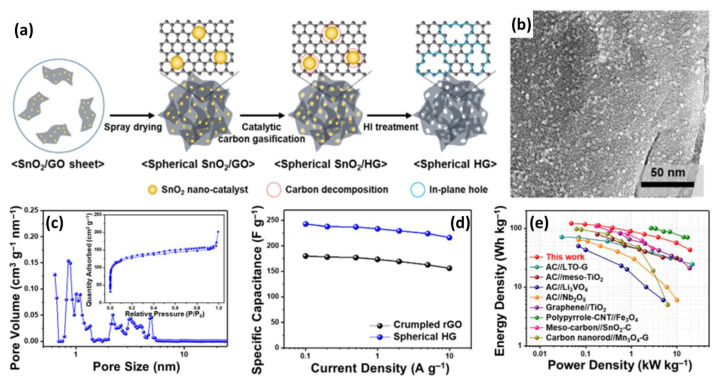
(**a**) Schematic of spherical HG preparation using catalytic carbon gasification; (**b**) TEM image and (**c**) N_2_ adsorption/desorption isotherm (inset) and pore size distribution of spherical HG; (**d**) Rate capabilities for spherical HG and crumpled rGO; (**e**) Ragone plots for the LICs composed of spherical HG and spherical LTO/HG composite. (Reprinted with permission from Ref. [104]. Copyright 2019 Elsevier.).

Catalytic carbon gasification is another method to prepare porous carbon materials. For example, Jeong et al. fabricated holey graphene (HG) with abundant in-plane pores using this method [104]. The synthesis process of HG is displayed in Figure 6a. Firstly, catalytic metal oxide (SnO_2_) nanoparticles were uniformly deposited onto the graphene oxide sheets via an aqueous-solution-based regular deposition process. Then, the SnO_2_/GO suspension was spray-dried to obtain a spherical composite, which was heated to induce the selective decomposition of the graphene adjacent to the SnO_2_ nanoparticles. It is noteworthy that the temperature should be carefully adjusted to be lower than the carbon combustion temperature in the catalytic carbon gasification process. Finally, the spherical SnO_2_/rGO was refluxed in HI to etch the catalysts and to reduce GO. The as-prepared HG has a spherical morphology and porous structure with rich in-plane holes of about 5 nm corresponding to the metal oxide size (Figure 6b). The N_2_ adsorption/desorption isotherm of HG indicates the existence of micropores and smaller mesopores (Figure 6c). This result can be further verified by the pore size distribution curve (inset of Figure 6c), which demonstrates that HG has micropores of 1nm and some mesopores of 2–6 nm. Due to its highly porous structure, HG shows much higher capacity than that of crumpled rGO prepared from GO by heat treatment (Figure 6d). As expected, LICs constructed using HG as a cathode and LTO/HG as an anode delivered a maximum energy density of 117.3 Wh kg^−1^, which still remains at 43.1 Wh kg^−1^ even at an extremely high power density of 19.7 kh kg^−1^ (Figure 6e). The outstanding electrochemical performance can be attributed to the good match of capacity and rate between the capacitor-type cathode and the battery-type anode.

In summary, benefitting from the 3D continuous porous structure and inherently high conductivity of graphene, pure graphene-based porous materials could deliver high capacity and superb rate capability and thus achieve high energy and power densities and cycling stability when used as a cathode in LICs, as summarized in Table 3. However, they suffer from the drawback of high cost and face the challenge of large-scale production. Hence, it is of great significance to develop cost-effective graphene-based porous materials with novel strategies, such as by forming composites with other low-cost materials.

## 4. Graphene-Based 3D Composites as Cathode Materials

Benefitting from the layered structure, graphenes are good 2D building blocks for constructing 3D porous composites [107]. Elaborately designed 3D graphene-based composites have a well-interconnected porous microstructure, high elasticity and mechanical strength, excellent chemical and electrochemical stability and high conductivity, making them superior candidates as cathodes for LICs [108,109]. In addition, the high cost of graphene largely inhibits its commercial applications, prompting researchers to fabricate graphene-based porous composites with other materials [110].

### 4.1. Grahene@Porous Carbon-Based 3D Composites

#### 4.1.1. Grahene@Non-Doped Porous Carbon 3D Composites

**Figure 7:**
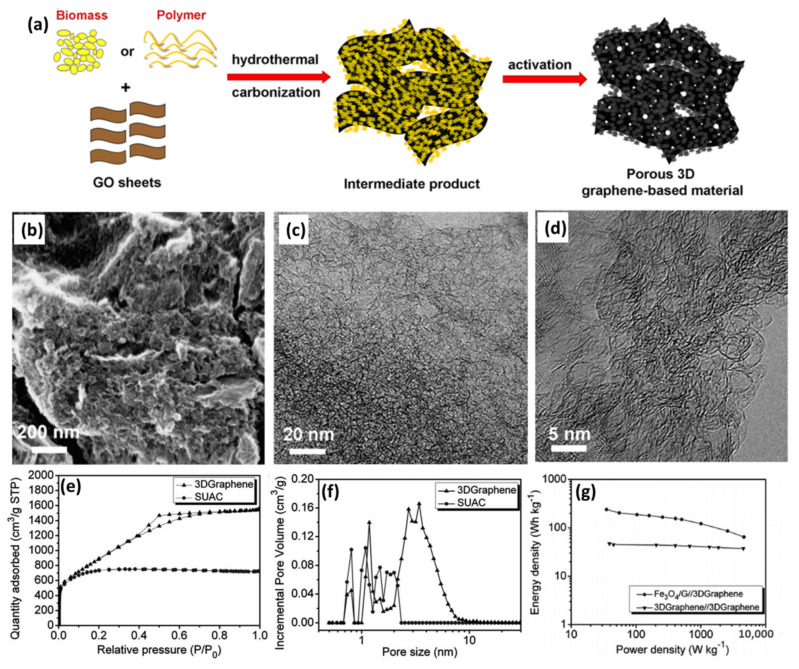
(**a**) Schematic of synthesizing porous 3D graphene-based materials; (**b**) SEM and (**c**,**d**) TEM images of 3DGraphene. (Reprinted with permission from Ref. [24]. Copyright 2013 Springer) (**e**) N_2_ adsorption–desorption isotherms and (**f**) pore size distribution of sucrose-based 3DGraphene and pure sucrose-based AC as contrast; (**g**) Ragone plots of the Fe_3_O_4_/G//3DGraphene LICs and 3DGraphene//3DGraphene supercapacitors. (Reprinted with permission from Ref. [74]. Copyright 2013 Royal Society of Chemistry.).

Chen’s group designed a simple, low-cost and green but very efficient approach to prepare 3D graphene-based porous materials (3DGraphene) with GO sheets and biomass or polymers (such as phenolic resin, sucrose and polyvinyl alcohol) using standard industry steps of in situ hydrothermal polymerization/carbonization and KOH activation (Figure 7a) [24]. The graphene sheets derived from GO could effectively block the stacking of the AC precursor generated from the matrix carbon sources during the hydrothermal reaction, and thus thinner and smaller AC particles are formed with more pores in the next chemical activation step. The activated products with the optimized ratio show a sponge-like morphology, highly porous structure and consist of mainly defected/wrinkled single-layer graphene sheets in the dimensional size of a few nanometers (Figure 7b–d). All the 3DGraphene materials have an ultrahigh SSA of over 3000 m^2^ g^−1^ and excellent bulk conductivity (up to 303 S m^−1^) because of the introduction of graphene. Taking the sucrose-based 3DGraphene, for example, it achieves an ultrahigh SSA of 3355 m^2^ g^−1^ with a well-controlled pore size of primarily 2–6 nm for the samples of optimized ratio (Figure 7e,f), making it an ideal capacitor-type material for LICs. By integrating it with an Fe_3_O_4_/graphene anode, the as-prepared LICs demonstrate a maximum energy density and power density of 204 Wh kg^−1^ at 4600 W kg^−1^, respectively, which is much higher than the 3DGraphene//3DGraphene symmetric supercapacitor (Figure 7g) [74]. Later, the same group also reported high-performance LICs by paring 3DGraphene with graphene-inserted LTO [111], flash-reduced graphene oxide [41] or graphene-modified phenolic resin-derived carbon [112], all of which displayed high energy and power densities and excellent cycling stability. These outstanding results are attributed to the graphene-based 3D porous carbon materials and the synergistic effect of the cathode and anode. The electrochemical performance has been significantly improved due to the introduction of graphene.

#### 4.1.2. Graphene@Doped Porous Carbon 3D Composites

**Figure 8:**
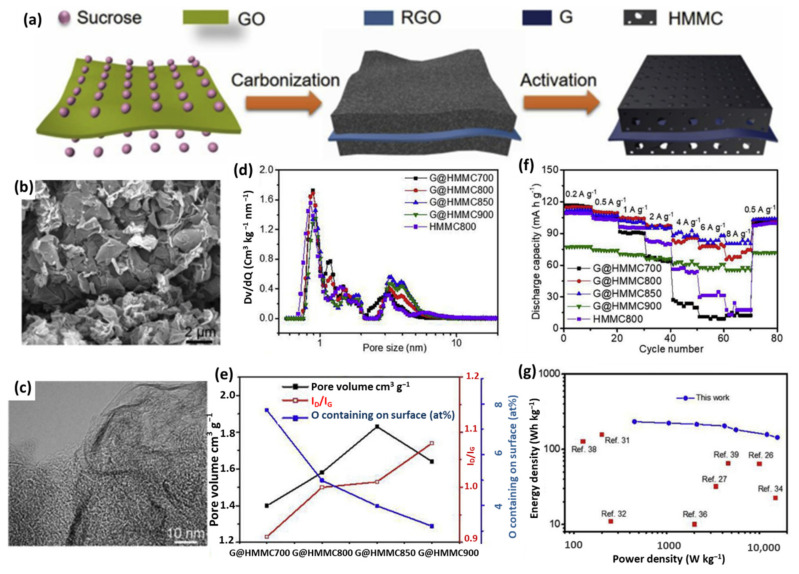
(**a**) Schematic of fabrication process of G@HMMC; (**b**) SEM and (**c**) TEM images of G@HMMC850; (**d**) PSD, (**e**) the pore volume, O contained on surface, and I_G_/I_D_ values and (**f**) rate performances of G@HMMC materials; (**g**) Ragone plot of G@HMMC-based LIC compared with other works. (Reprinted with permission from Ref. [116]. Copyright 2018 Elsevier.).

Although the capacity of porous graphene-based cathodes is largely improved compared with AC, there is still a long way to go to meet the ever-growing demand of materials with high capacity. Several simple but effective methods have been adopted to further increase the capacity of capacitor-type electrodes, such as designing novel structures and/or doping with heteroatoms [113,114,115]. Li et al. reported a sandwich-like graphene@hierarchical meso-/micro-porous carbon (G@HMMC) with functional oxygen containing groups through a facile carbonization and chemical activation procedure (Figure 8a) [116]. As displayed by the SEM and TEM images in Figure 8b,c, G@HMMC is composed of wrinkled multilayer nanosheets with a sandwich-like porous structure and the 2D structure of graphene is well retained after activation. The hierarchical porous structure of G@HMMC is consistent with the PSD characterization, which is mainly meso-/micropores (Figure 8d). For the optimized sample (G@HMMC850), the highest SSA of 2674.6 m^2^ g^−1^ and the largest pore volume of 1.67 cm^3^ g^−1^ were obtained (Figure 8e). Coupling with a moderate oxygen content that could provide extra capacity by fast redox reaction and increase the wettability with electrolytes, G@HMMC850 delivers a high specific capacity of 112 mAh g^−1^ at 0.2 A g^−1^ and remains around 90 mAh g^−1^ at 8.0 A g^−1^, making it an advanced capacitor-type material (Figure 8f). As expected, LICs using G@HMMC850 as a cathode achieve an ultrahigh energy density of 233.3 Wh kg^−1^ at 450.4 W kg^−1^, which still keeps 143.8 Wh kg^−1^ at an extremely high power density of 15.7 kW kg^−1^ (Figure 8g). The high performance can be ascribed to the hierarchical meso-/microporous structure and the oxygen-containing groups of the graphene-based sandwich-like 3D material, which provides sufficient active sites for adsorption/reactions and facilitates fast electron and ion transportation. Additionally, other graphene-based 3D porous composites using phenolic resin- or pitch-based precursors are prepared to serve as capacitor-type electrodes [112,117].

Additionally, N-doped graphene-based porous composites are considered to be promising candidates because of their excellent conductivity and increased capacitance contributed by the electrical double layer and redox reactions [118]. For example, Fan et al. prepared nitrogen-doped graphene-based aerogel composites (NGA) through a sol–gel polymerization of resorcinol, formaldehyde and melamine in the presence of GO, and then activation with KOH [98]. After carbonization and activation, the obtained NGA showed a loosely packed morphology with a much thinner laminar structure than non-doped samples due to the nitrogen dopant, but they still retained the 3D interconnecting network. This structure provides NGA with much easier electrolyte ion diffusion while keeping high electrical conductivity, making it an excellent rate capacitor-type electrode. As expected, the assembled LICs with NGA as the cathode and LTO as the anode provided maximum energy and power densities of 70 Wh kg^−1^ and 8 kW kg^−1^, respectively.

**Figure 9:**
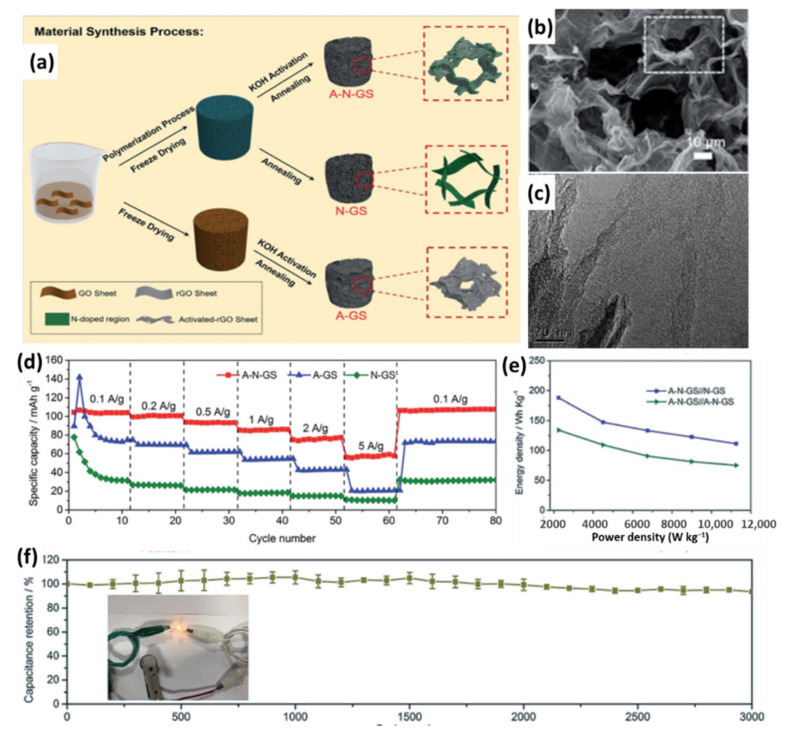
(**a**) Schematic illustration of the synthesis of A-N-GS, A-GS and N-GS; (**b**) SEM and (**c**) TEM images of A-N-GS; (**d**) Rate performance of A-N-GS, A-GS and N-GS; (**e**) Ragone plots of A-N-GS//N-GS and A-N-GS//A-N-GS LICs; (**f**) Cycle performance of A-N-GS//N-GS LICs. (Reprinted with permission from Ref. [120]. Copyright 2019 Royal Society of Chemistry.).

Polyaniline, as one of the most attractive nitrogen-rich polymers, is typically used as a nitrogen dopant precursor for preparing N-doped carbons due to the advantages of simple synthesis, good conductivity and a high content of nitrogen [119]. Wang et al. designed a high-voltage LIC with all-graphene-based materials prepared by tuning the synthetic chemistry [120]. As presented in Figure 9a, the N-doped capacitor-type cathode (A-N-GS) was fabricated through the polymerization of aniline in the presence of GO and then chemically activated by KOH. The battery-type anode (N-GS) was prepared with a similar method except for the activation treatment. An activated graphene sheet (A-GS) without N doping was also prepared and compared with A-N-GS. The as-obtained A-N-GS exhibited a 3D porous structure and sheet-like morphology with a lot of wrinkles on the surface (Figure 9b). A lot of micropores can be observed, which were caused by the combined effect of polyaniline decomposition and KOH activation (Figure 9c). Benefiting from the hierarchically porous structure with continuously interconnected channels, highly conductive networks and heteroatom doping, the A-N-GS demonstrates a capacity of 116 mAh g^−1^ at 0.1 A g^−1^ and still retains 56.8 mAh g^−1^ at 5 A g^−1^, which is a much higher capacity than that of A-GS and N-GS (Figure 9d). Consequently, LICs constructed with an A-N-GS cathode and an N-GS anode achieved a high energy density of 187.9 Wh kg^−1^ and power density of 11.25 kW kg^−1^, considerably superior to those of symmetrical devices based on A-N-GS//A-N-GS (Figure 9e). Furthermore, the A-N-GS//N-GS LIC demonstrated excellent long-term cycling stability with a capacity retention of 93.5% after 3000 cycles at 2 A g^−1^ (Figure 9f). Based on the above reports, the capacity and rate capability of the capacitor-type cathode could be effectively improved by heteroatom doping because of the rich porosity, increased conductivity, better electrolyte wettability and extra capacitance provided by fast redox reaction.

### 4.2. Graphene/Nanostructured Material 3D Composites

**Figure 10:**
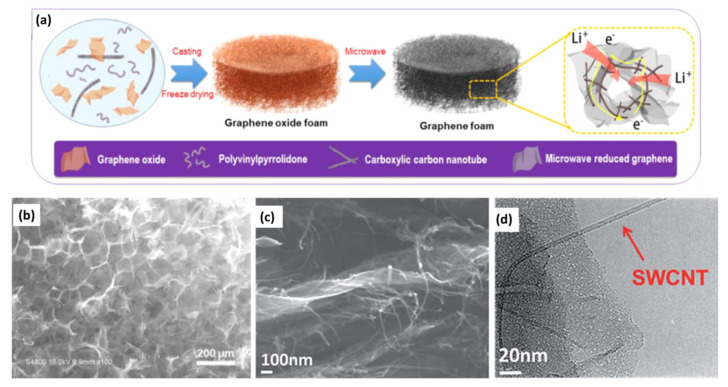
(**a**) The schematic illustration of preparation of GF and (**b**) SEM image of GF. (Reprinted with permission from Ref. [126]. Copyright 2020 Elsevier.) (**c**) SEM and (**d**) TEM images of SG. (Reprinted with permission from Ref. [127]. Copyright 2017 Royal Society of Chemistry.).

In the previous reports, conductive extra spacers (such as CNTs, ACs or conductive polymers, etc.) were usually introduced to prevent the restack of graphene-based materials by forming 3D composites, which leads to high SSA and/or electrical conductivity, and thus excellent specific capacitance and rate capability could be obtained [80,121]. Similar strategies have been adopted to prepare high-performance capacitor-type electrodes for LICs [122,123,124,125]. In such cases, the serious aggregation or restacking of graphene could be suppressed so that the effective SSA could be enhanced. On the other hand, the spacers could also provide additional capacity through ion adsorption/desorption. For example, Wang et al. synthesized a durable and self-supporting graphene foam (GF) by microwave reduction of a polyvinyl pyrrolidone-stabilized GO/carboxylic CNT composite (Figure 10a) [126]. As displayed in Figure 10b, a reticulum-like porous structure of the as-prepared GF was generated after the rapid reduction. More importantly, CNTs serving as the spacer not only effectively prevent the restacking of reduced GO but also form a 3D conductive network. Benefiting from the highly porous structure and excellent electrical conductivity of GF, the assembled LICs with a porous LTO on CNT film as an anode exhibited an energy density of 101.8 Wh kg^−1^ (at a power density of 436.1 W kg^−1^) and a capacitance retention of 84.8% after 5000 cycles. Sun et al. fabricated a single carbon nanotube (SWCNT) and graphene composite (SG) by inserting the SWCNT into the space between the graphene nanosheets [127]. As shown in the SEM and TEM images of the SG composite (Figure 10c,d), SWCNTs were successfully inserted and homogeneously distributed between the graphene layers, which resulted in a highly 3D porous structure and an improved overall electrical conductivity. Therefore, the SG composite can effectively prevent the restacking of graphene and provide sufficient active sites for ion adsorption/desorption, making it a favorable capacitor-type electrode for LICs.

**Figure 11:**
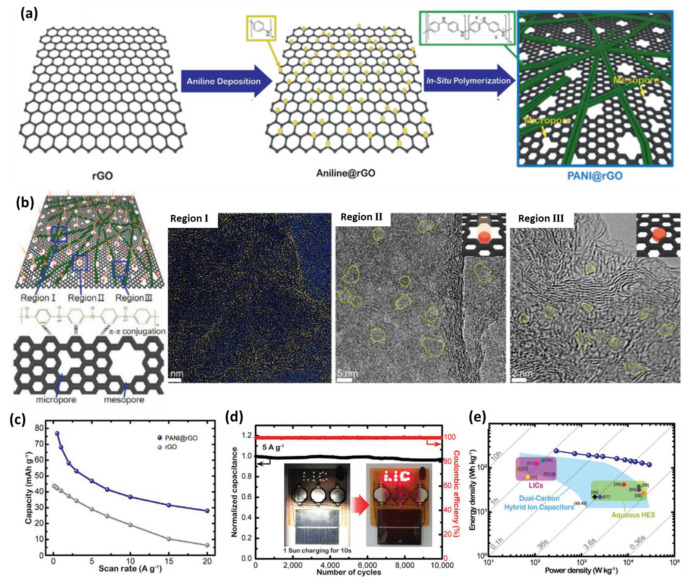
(**a**) Schematic illustration of in situ polymerized polyaniline chain web cathode units integrated into rGO sheets to form the PANI@rGO cathode; (**b**) Schematic images of ion storing of PANI@rGO cathode and π–π conjugation between the PANI chain and rGO sheet and HRTEM images of region I (conjugated PANI, temperature mode), region II (mesopores), region III (micropores); (**c**) Rate capabilities of PANI@rGO and rGO; (**d**) Cycling stability and (**e**) Ragone plot of MoO_2_@rGO//PANI@rGO LICs. (Reprinted with permission from Ref. [128]. Copyright 2020 Wiley-VCH.).

In addition, conductive polymers such as polyaniline (PANI) can also be applied as spacers to inhibit the restacking of graphene sheets considering their unique merits of high conductivity, excellent flexibility and high capacity derived from fast redox reaction. Ock et al. proposed a high-capacity and high-rate PANI@rGO and used it as a capacitor-type electrode [128]. PANI@rGO was synthesized through depositing aniline on the surface of GO sheets and then forming a chain-integrated rGO-based composite after in situ polymerization and cross-linking of PANI (Figure 11a). The obtained PANI@rGO has a layered morphology and multi-porous structure with PANI forming an interconnected network on the rGO sheets by π–π conjugation (Figure 11b). As demonstrated in the HRTEM images, the pores of rGO are mainly micropores and mesopores generated by removing the oxygen-containing groups during the reduction process (Figure 11b). Based on its outstanding characteristics, PANI@rGO showed a capacity of about one time higher than that of rGO at all current densities (Figure 11c). The improved capacity and rate performance could be ascribed to a non-Faradaic reaction with extremely rapid adsorption/desorption of electrolyte ions at the electrode/electrolyte interface as well as the extra capacity provided by the fast redox reactions. By integrating the PANI@rGO with a rGO-coated MoO_2_ anode, the full LIC cell exhibited an ultralong cycling life with 96% capacity retention after 10,000 cycles at 5 A g^−1^ (Figure 11d). Moreover, the as-fabricated LIC showed a maximum energy density of 241.7 Wh kg^−1^, which still maintained 117.8 Wh kg^−1^ at an extremely high power density of 28.75 kW kg^−1^ (Figure 11e). The enhancement of electrochemical performance can be roughly ascribed to the improved electrical conductivity and high SSA because of the introduction of extra spacers to prevent the aggregation of graphene.

### 4.3. High-Density Graphene-Based 3D Composites

**Figure 12:**
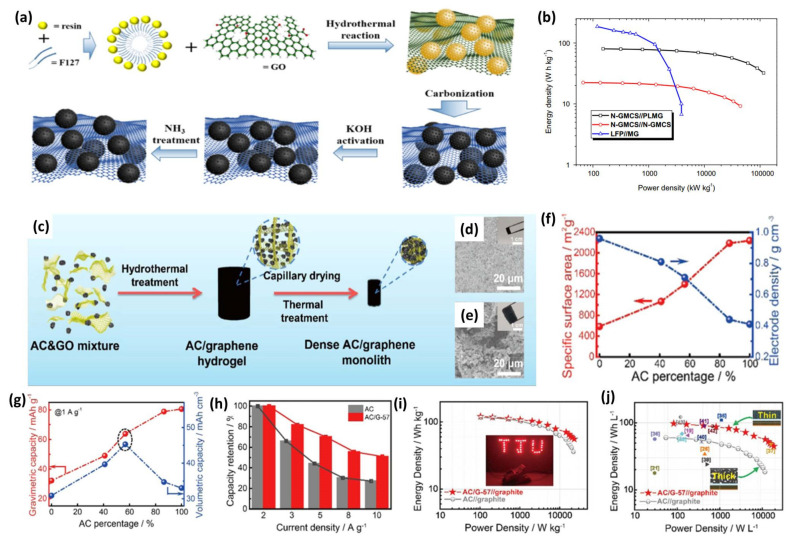
(**a**) Schematic of the synthesis of the NGMCS nanocomposite; (**b**) TEM image of the N-GMCS nanocomposite. (Reprinted with permission from Ref. [130]. Copyright 2015 Elsevier.) (**c**) Schematic of the AC/G composite monolith fabrication process; SEM images of (**d**) the capillary drying-induced dense AC/G monolith and (**e**) the freeze-drying-induced AC/G hydrogel; (**f**) The SSA and electrode density variations of the AC/G composites with the percentage of AC; (**g**) The relationship between the gravimetric/volumetric capacity and the percentage of AC in the AC/G composites; (**h**) Comparison of the capacity retention of pure AC and AC/G-57 electrodes at various current densities; Ragone plot of the AC/G-57//graphite LIC compared with the AC//graphite LIC based on (**i**) mass and (**j**) volume. (Reprinted with permission from Ref. [131]. Copyright 2019 Elsevier.).

Commonly, carbon-based cathode materials suffer from low packing/tap density because of their highly porous structure, and this is more serious for nanostructured carbon materials (e.g., graphene or CNTs), which always leads to inferior volumetric energy density [129]. Therefore, developing materials with high packing/tap density is of great significance for fabricating high energy density LICs. For instance, Huang et al. demonstrated a novel nitrogen-enriched mesoporous carbon nanosphere/graphene (N-GMCS) composite with high packing density [130]. The preparation process of N-GMCS includes the following steps (Figure 12a). Initially, mesoporous carbon nanospheres were formed on the graphene oxide substrate through in situ polymerization of phenolic resin under a hydrothermal reaction and subsequent carbonization. After the intermediate product was further activated by KOH and doped with abundant nitrogen functionality by NH_3_ treatment, the final product was obtained. N-GMCS possesses a hierarchical porous structure, 3D conductive network as well as high packing density (0.6 g cm^−3^), making it a promising capacitor-type electrode for high-energy devices. As expected, LICs with N-GMCS as the cathode delivered maximum energy and power densities of 80 Wh kg^−1^ and 352 kW kg^−1^, respectively (corresponding to 66.7 Wh L^−1^ and 292 kW L^−1^) (Figure 12b).

Recently, Yang and coworkers designed a highly dense but porous activated carbon/graphene (AC/G) composite as the capacitor-type cathode for high gravimetric/volumetric energy density LICs [131]. The typical preparation of AC/G composites involves a process of hydrothermal reaction, capillary drying and high-temperature annealing (Figure 12c). As clearly demonstrated in the SEM images and photographs in Figure 12d, the volume of AC/G hydrogel and the voids between AC microparticles are significantly reduced to obtain a dense AC/G monolith after the capillary drying process, illustrating that a compact microstructure is formed. By contrast, the AC/G-FD monolith prepared through the freeze-drying process keeps almost the same size and appearance as the intermediate hydrogel with a loose morphology (Figure 12e), which strongly shows the advantages of capillary drying in preparing high-density electrodes. The highly dense but porous characteristic of AC/G composites can be verified by the results of SSA and electrode density (Figure 12f). For example, AC/G-57 has an SSA of 1402 m^2^ g^−1^ and its electrode density is still as high as 0.71 g cm^−3^, which is around 173% of that of AC. Hence, although AC/G-57 shows relatively lower gravimetric specific capacity compared with pristine AC, it has the largest volumetric specific capacity of 45 mAh cm^−3^, which is 137% of AC and 147% of HPGM, respectively (Figure 12g). Furthermore, AC/G-57 shows a higher capacity than that of AC, especially at high current density, which can be attributed to the fast and effective ion and electron conductive network formed by the 3D graphene skeleton (Figure 12h). Consequently, LICs with AC/G-57 as the cathode show superior electrochemical performance compared to pristine AC-based LICs in both gravimetric and volumetric energy densities (Figure 12i,j). A similar strategy has been applied to prepare other 3D graphene-based composites as capacitor-type electrodes for advanced LICs. Liu et al. fabricated a high tap density graphene material from a few-layer graphene/GO composite for LICs via a simple blast drying [132]. The obtained cathode shows an apparent volume shrinkage and has an extremely high tap density of 0.7 kg L^−1^. More importantly, the mass loading of the electrode can reach as high as 13.5 mg cm^−2^, resulting in a reversible capacity of 136.4 mAh g^−1^ at 0.1 A g^−1^ and maintaining about 63% at 200 C. There is no doubt that high volumetric energy density is equally as important as the gravimetric energy density, which is one of the critical factors influencing the commercial applications of LICs. Hence, developing graphene-based cathodes with high tap density is of great importance and compact 3D graphene-based materials should be elaborately designed.

Based on the above discussion, graphene-based 3D composites show obvious advantages compared with reduced GO and pristine porous graphene, as summarized in Table 4. Reduced GO usually has unsatisfactory energy and power densities resulting from their relatively low SSA and poor electrical conductivity because of serious aggregation/restacking of graphene nanosheets and the unrecovered conductive network, while pristine porous graphene suffers from high cost and/or complicated synthesis procedures. Hence, forming composites with other highly conductive materials to prepare graphene-based 3D porous materials is a facile but cost-effective strategy. Future research should focus on designing materials with well-controlled porosity, tunable microstructure and morphology and high packing density.

## 5. Summary and Outlook

LICs possess the merits of a high energy density, high power density, large operation voltage, wide working temperature range and long cycling stability, and thus are considered to be one of the ideal energy storage devices to overcome the disadvantages of batteries and supercapacitors. Although LICs have the potential to bridge the gap between batteries and supercapacitors, their electrochemical performance still fails to reach the requirements of commercial applications. Especially, the unsatisfactory energy density of state-of-the-art LICs is one of the main obstacles preventing their applications, which is mainly due to the low capacity of the capacitor-type electrode and the kinetics and capacity imbalances between the cathode and anode. For instance, traditional capacitor-type materials such as AC suffer from inferior specific capacity and poor rate performance because of a relatively low SSA and electrical conductivity. Hence, developing high-capacity capacitor-type materials is significantly important to obtain high-energy LICs. Thanks to their unique 2D structure, high electrical conductivity, large SSA, tunable surface chemistry and excellent chemical and electrochemical stability, graphene-based cathode materials have demonstrated remarkable achievements in LICs. In this review, a systematic summary of graphene-based capacitor-type materials has been presented. It could be easy to conclude from the above results that graphenes, either as active materials or as building blocks to form composites with other materials, have shown great potential in capacitor-type cathode candidates for LICs.

Nevertheless, the performance of the current graphene-based capacitor-type electrodes is still far from the practical requirements. Moreover, other remaining challenges related to battery-type anodes, the production of graphene-based materials, volumetric performance, electrolytes, performance imbalance between the anode and cathode and pre-lithiation technologies should be given more attention, too. Thus, the following several aspects should be considered to overcome the present challenges and to realize the commercialization of LICs:

(1) High-capacity capacitor-type materials are urgently needed. To match the high capacity of battery-type anodes, developing cathode materials with improved capacity is the top priority. The cathode stores energy through a physical adsorption/desorption process at the electrolyte/electrode interface, which leads to a fast charge/discharge rate. On the other hand, the non-Faradaic energy storage mechanism also results in low capacity because it is critically influenced by the SSA. A high SSA could provide more active sites for ion adsorption and, to a certain extent, the capacity is raised with the increase in the SSA. However, it should be pointed out that not all the surface can be accessible by the electrolyte ions [134]. Therefore, the morphology, pore size and surface chemistry of graphene-based cathode materials should be carefully regulated to increase the effective surface area and in turn enhance their capacity. In addition, heteroatom doping and porosity engineering should also receive enough attention to obtain high-capacity and high-rate capacitor-type cathodes. Doping can not only provide extra capacity by fast redox reactions but enhance the electrical conductivity, while rational and tunable porosity is beneficial for the electrolyte ions’ diffusion, together resulting in excellent rate capability.
(2) The preparation of graphene-based cathode materials at low cost is one of the critical factors for large-scale applications. Although pure graphene-based porous materials can serve as outstanding capacitor-type electrodes due to their high electrical conductivity and large SSA, the high cost impedes their further commercial utilization. Forming composites with other materials is a facile but feasible method to solve this problem, such as biomass or polymer/graphene hydrogel-derived graphene-based 3D porous materials initially proposed by Chen’s group [24]. In such cases, the overall cost of the cathode materials can be largely reduced but the high conductivity and porous structure can be kept.
(3) Developing battery-type materials with a high rate and long-term stability is another big challenge but imperative issue. High-capacity anodes ensure the high energy of the full cell. However, the high energy density of state-of-the-art LICs could only be realized at the cost of low power output because of the sluggish redox reaction and/or inferior electrical conductivity of battery-type anodes. Hence, designing nanostructured materials with tunable porosity and compositing with highly conductive materials are always applied to achieve high-rate anodes. These strategies could help to reduce the capacity and kinetics imbalances between the cathode and anode, leading to improved energy and power densities [12]. With the booming of lithium metal batteries, Li metal anodes have also been applied as the battery-type electrode to develop high-energy LICs [131,135]. In this circumstance, elaborate surface coating or electrolyte regulation is needed to suppress lithium dendrite growth to avoid safety accidents [136,137].
(4) The volumetric performance of graphene-based materials should receive more attention for commercial applications. Graphene-based cathode materials have shown enhanced gravimetric capacity compared with commercial AC. However, their large SSA and highly porous structure result in a low taping density and consequently low volumetric energy density, which is a big obstacle for practical utilization. In addition, more binders and solvents are needed in the electrode fabrication process because of the highly porous nanostructured carbon materials, increasing the manufacturing cost and decreasing the energy density. Thus, the porosity and taping density should be well balanced. To achieve high volumetric energy density at a low cost, future research can focus on forming graphene-based 3D composites by using capillary drying or rapid drying processes [131,132].
(5) Advanced electrolytes with a wide working voltage and high safety are also needed. Currently, typical LICs commonly adopt organic electrolytes to achieve a high operation voltage. However, they suffer from safety issues associated with volatility, flammability and toxicity. Hence, other novel electrolytes have been explored. For example, ionic liquids are regarded as a promising alternative to the organic electrolyte owing to their large working voltage, high conductivity and excellent thermal stability without risk of catching fire [134,138]. Recently, “Water-in-Salt” electrolytes have drawn tremendous interest as they inherit the safety advantage of aqueous electrolytes while keeping the high working voltage of organic electrolytes [139,140,141,142].
(6) The decomposition of electrolytes should not be ignored. Generally, electrolyte decomposition on the cathode and anode takes place during the charge/discharge process, especially at a high working voltage, resulting in gas emission, impedance increase and low energy conversion efficiency. At the anode side, this phenomenon can be largely restrained by forming a stable solid–electrolyte interface (SEI) during the first several cycles. However, an effective strategy to suppress the electrolyte decomposition at the cathode side is still absent [143]. Fortunately, Li et al. proposed a preliminary electrochemical coating process to form a well-formed protective layer on a graphene-based cathode [144]. The protective layer could block the electron flow from the cathode to the electrolyte and thus terminate the decomposition. This may be a possible and promising solution to obtain high-voltage and long-durability LICs. Furthermore, some in situ and ex situ characterization technologies could be applied to investigate the decomposition mechanism of electrolytes, the degradation and/or evolution of the electrode surface and electrode/electrolyte interface and the composition of decomposed products [145,146,147]. It is expected that these in situ and ex situ tools will help us to understand the beneath decomposition mechanism and find an effective protection method.
(7) Besides the above discussion, other critical issues should also be solved before industrial-level production and widespread applications. First of all, feasible pre-lithiation technology should be developed because the anode, especially nanostructured materials with a large SSA and rich porosity, would consume a large amount of lithium ions when forming a stable SEI [148,149]. Well-controlled pre-lithiation could largely improve the structural stability of the electrode, enhance the reversible capacity and working voltage of the full cell and reduce the resistance, which thus increases the energy and power densities and cycling life. However, current pre-lithiation technologies such as internal or external short circuit [150,151] or using lithium-containing compounds [152,153,154] are either unsafe, time-consuming or inefficient. Hence, high-efficiency pre-lithiation methods are highly needed. Benefiting from well-developed LIBs and SCs, the design strategies, assembly technology and components (conductive additives, binders and shell) could be easily transferred to LICs [133]. More importantly, special attention should be given to thermal management, which is exceptionally critical for the safe and efficient operation of LICs, especially at high and low temperatures [155].
(8) Additionally, considering the limited and uneven distribution of lithium resources, other metal-ion capacitors are drawing increasing attention and have recently become the research hotspot. In particular, monovalent ion systems (i.e., sodium-ion capacitors and potassium-ion capacitors) are the most promising alternatives to LICs because they have a similar cell configuration and energy storage mechanism as well as abundant resources [156,157]. However, like LICs, Na/K-ion capacitors also suffer from safety problems derived from the use of organic electrolytes and the formation of highly reactive metal dendrites. Recently, multivalent ion systems are drawing more interest due to the merits of providing twice or triple the amount of electrons per unit of active materials as well as being less sensitive to air and water, rendering them low-cost, high-energy and safe devices [158,159]. These novel systems are still in the early stage and more efforts should be devoted to preparing advanced electrode materials, developing suitable electrolytes and investigating the energy storage mechanism.

In conclusion, LICs have witnessed tremendous progress during the last decade, especially in electrode materials. Their development steps are speeding up and large-scale application will be realized in the near future. Without a doubt, graphene-based nanomaterials could play a vital role in achieving high performance due to their outstanding electrochemical properties.

## Figures and Tables

**Table 1:** The electrochemical performances of various energy storage systems.

Energy Storage Systems	Anode//Cathode	Electrolyte	Voltage (V) ^a^	Energy Density (Wh kg^−1^) ^b^	Power Density (W kg^−1^) ^c^	Cycling Life
LABs	Pb//PbO_2_	H_2_SO_4_ aqueous solution	2	30–50	<1000	<800
NiMHBs	Metal hydride//Ni(OH)_2_	KOH aqueous solution	1.2	40–60	~1000	<1000
LIBs	Graphite//Lithium-based compounds ^d^	LiPF_6_ in organic solution	3.6–4.35	150–300	<1000	<5000
EDLCs	AC//AC	(CH_3_CH_2_)_4_NBF_4_ in acetonitrile	2.7–3.0	5–10	>10,000	>100,000
LICs	Battery-type anode//Capacitor-type cathode	Lithium salts in organic solution	3.0–4.5	20–100	1000–10,000	>10,000

Note, ^a^: maximum working voltage; ^b^ and ^c^: based on the mass of full cell; ^d^: LiFePO_4_, LiCoO_2_, LiMn_2_O_4_ or LiNi_x_Co_y_Mn_z_O_2_ (x + y + z = 1).

**Table 2:** Properties of reduced graphene oxide cathode and the performances of LICs.

Cathode//Anode	Electrode Preparation	Capacity of Cathode (mAh g^−1^)	Electrolyte	Cell Voltage (V)	Maximum Energy Density (Wh kg^−1^)	Maximum Power Density (kW kg^−1^)	Cycling Stability	Ref.
URGO//graphite	Reduced by urea	35	1 M LiPF_6_ in EC/DEC	2.2–3.8	106	4.2	~100% at 1000	[85]
TRGO//LTO	Reduced by trigol	58	1 M LiPF_6_ in EC/DEC	1–3	45	3.3	~100% at 5000	[88]
EG-GO//Li	Hydrothermal reduction	172	1 M LiPF_6_ in EC/DEC/DMC	2–4.5	240	53.5	~100% at 3000	[84]
Graphene grass//TNO	Hydrothermal reduction	63.2	1 M LiPF_6_ in EC/DMC	0–3	74	7.5	81.2% at 3000	[92]
PRGO//N-CNPipes	Thermal annealing	171	1 M LiPF_6_ in EC/PC	0.01–4	262	9.0	91% at 4000	[89]
Graphene hydrogel//TiO_2_ NBA	Hydrothermal reduction	52	1 M LiPF_6_ in EC/DMC	0–3.8	82	19	73% at 600	[90]
PGM//LTO/C	Hydrothermal reduction	66	LiPF_6_	1–3	72	8.3	65% at 1000	[91]

**Table 3:** Properties of pure graphene-based cathode and the performances of LICs based on them.

Cathode//Anode	Electrode Preparation	Capacity of Cathode (mAh g^−1^)	Electrolyte	Cell Voltage (V)	Maximum Energy Density (Wh kg^−1^)	Maximum Power Density (kW kg^−1^)	Cycling Stability	Ref.
SNMG//LTO	CVD	112	1 M LiPF_6_ in EC/DEC	0–4	86.2	7.4	87% at 2000	[96]
PGBs//LTO	CVD	92	1 M LiPF_6_ in EC/DEC	0–4	120	8.04	83.7% at 2000	[97]
CG	Template-guided	212.3 F g^−1^	1 M LiPF_6_ in EC/DEC/DMC	1–4	121	18	87% at 2000	[105]
HG	Catalytic carbon gasification	97.2	1 M LiPF_6_ in EC/DEC	1.5–3	117.3	19.7	81.7% at 2000	[104]
a-MEGO//graphite	Chemical activation	125	1 M LiPF_6_ in EC/DEC	2–4	147.8	/	/	[101]
a-NGA//LTO	Chemical activation	76	1 M LiPF_6_ in EC/DEC/DMC	1–3	70	8.0	64% at 10,000	[98]
AGF	Chemical activation	93 F g^−1^	1 M LiPF_6_ in EC/DEC	0–3	53	2.09	89% at 3000	[102]
PG	Chemical activation	69	1 M LiPF_6_ in EC/DEC/DMC	0.01–4.2	135.6	21	65% at 3000	[103]
NGF-0//NGF-2	Magnesiothermic combustion synthesis	82	1 M LiPF_6_ in EC/DEC/DMC	1–4	151	49	87% at 10,000	[106]

**Table 4:** Properties of cathodes using graphene-based 3D composites and the performances of LICs.

Cathode//Anode	Electrode Preparation	Capacity of Cathode (mAh g^−1^)	Electrolyte	Cell Voltage (V)	Maximum Energy Density (Wh kg^−1^)	Maximum Power Density (kW kg^−1^)	Cycling Stability	Ref.
PC-75//MnO@C	Chemical activation	50	1 M LiPF_6_ in EC/EMC/DMC	0.1–4	117.6	10.25	76% at 3000	[117]
N-GMCS//graphite	Chemical activation	/	1 M LiPF_6_ in EC/DEC	2.2–4.2	66.7 Wh L^−1^	292 kW L^−1^	93.1 at 3000	[130]
3DGraphene// Fe_3_O_4_/G	Chemical activation	/	1 M LiPF_6_ in EC/DEC/DMC	1–4	204	4.6	70% at 1000	[74]
3D PANI/GNSs//3D MoO_3_/GNSs	Chemical activation	67.8	1 M LiPF_6_ in EC/DEC	0–3.8	128.3	13.5	90% at 3000	[119]
GF//CNT@pLTO	Microwave oven irradiation	151.9 F g^−1^	1 M LiPF_6_ in EC/DMC	0–3.5	108.1	12.3	84.8% at 5000	[126]
rGO-CNT//lithiated rGO-CNT	Electrostaticspray deposition	72	1 M LiPF_6_ in EC/EMC	0.01–4.3	114.5	2.57	68.5% at 2000	[123]
SG//Li-SG	Reduced by hydrazine	137 F g^−1^	1 M LiPF_6_ in EC/DMC	0–4	222	/	58% at 5000	[127]
OAC/rGO//Si/C	Ball milling	140	1 M LiPF_6_ in DMC/FEC	2–4.5	141	10.3	78.9% at 1000	[124]
PANI@rGO// MoO_2_@rGO	In situ polymerization	/	1 M LiPF_6_ in EC/DEC	1.25–4.5	241.7	28.75	96% at 10000	[128]
G@HMMC//graphite	Chemical activation	112	1 M LiPF_6_ in EC/DEC/DMC	2–4.5	233.3	15.6	90.6% at 3000	[116]
AC/G//graphite	Hydrothermal Process and thermal treatment	45 mAh cm^−3^	1 M LiPF_6_ in EC/EMC/DMC	2–4.5	98 Wh L^−1^	19 kW L^−1^	98.9% at 3000	[131]
G/AC//G/SC	Self-propagating high-temperature synthesis	113.7F g^−1^	1 M LiPF_6_ in EC/DEC/DMC	1–4	151	18.9	93.8% at 10,000	[133]
